# Effect of miR-215 on the Expression of Tumor Suppressor Gene Rb1 in Retinoblastoma Cell Lines

**DOI:** 10.18502/ijph.v49i7.3583

**Published:** 2020-07

**Authors:** Liqin SHAO, Zhangxing SHENG, Yuefeng ZHU, Jianchao LI, Rufa MENG

**Affiliations:** 1. Department of Ophthalmology, Affiliated Hospital of Shaoxing University, Shaoxing, Zhejiang 312000, China; 2. Department of Ophthalmology, Xi’an Traditional Chinese Medicine Hospital, Xi’an, Shaanxi 710020, China; 3. Department of Ophthalmology, The Fifth Hospital of Shaoxing, Shaoxing, Zhejiang 312000, China

**Keywords:** miR-215, Retinoblastoma, Retinoblastoma (Rb)

## Abstract

**Background:**

Effect of miR-215 on the expression of tumor suppressor gene retinoblastoma (Rb)1 in Rb cell lines was investigated.

**Methods:**

A total of 128 patients were selected. The expression of miR-215 in cancer and adjacent healthy tissues of the 128 patients was detected by reverse transcription-quantitative PCR (RT-qPCR). HXO-Rb44 and Y79 cell lines were transfected with miR-215 analogs or miR-215 inhibitors, and the expression of Rb1 protein in the cell lines was detected by western blotting.

**Results:**

The expression of miR-215 in the adjacent healthy tissues of patients was significantly lower than that in cancer tissues (*P<*0.001). The expression of miR-215 in Y79 and HXO-Rb44 cells was significantly higher than that in APRE-19 cells (*P<*0.001). The expression of miR-215 in HXO-Rb44 cells was significantly higher than that in Y79 cells (*P<*0.001). The expression of miR-215 was statistically different from the degree of differentiation and nerve infiltration (*P<*0.05). The expression of Rb1 in cancer tissues was significantly lower than that in adjacent tissues (*P<*0.001), the expression of APRE-19 was significantly higher than that in Y79 and HXO-Rb44 cells (*P<*0.001), and the expression of Rb1 in HXO-Rb44 cells was significantly higher than that in Y79 cells (*P<*0.05). There was a negative correlation between miR-215 and Rb1 in the tissues of patients, and Rb1 expression decreased with the increase of miR-215 (r=-0.576, *P<*0.001).

**Conclusion:**

miR-215 is highly expressed in Rb cell lines, and is related to the clinicopathological features of this disease.

## Introduction

The incidence of retinoblastoma (Rb) in infants and children ranks second to that of leukemia. Rb is an embryonic malignant tumor of the retinal neuroepithelium. As the most common type of intraocular malignant tumor, Rb seriously affects children’s visual acuity and life (1,2). Rb frequently occurs in children 0–4 years of age, and the incidence is not related to race, sex or age (3). The degree of malignancy of Rb is usually high. When the children are admitted to the hospital, they are already at the middle and advanced stages of the disease, and have missed the best time for treatment. Traditional surgery, as well as radiotherapy and chemotherapy usually fail to provide satisfactory treatment outcomes, so the survival rate is low. In addition, most patients are diagnosed at advanced stages, and eye ball removal is usually necessary to ensure the safety of the children.

miRNAs are a class of endogenous non-coding small RNAs with a length of ∼19–25 bp. miRNAs increase the degradation and affect the translation of mRNA by binding to the 3′-UTR region to regulate gene expression at post-transcription level (4). MiR-215 is expressed in different tumors (5,6), and miR-215 performs its biological functions by affecting the proliferation, invasion and other aspects of the tumors (7). Rb1 expression is lowly expressed in Rb tissues, and is related to gene mutation and genetic modification and other molecular mechanisms (8). Rb1 is differentially expressed in a variety of tumors, such as breast cancer (9), pancreatic cancer (10), and lung cancer (11). However, the effects of miRNA on Rb1 expression in Rb cells, and its relationship with Rb is still unknown.

Therefore, we aimed to detect the expression of miR-215 in cancer tissues and adjacent healthy tissues, and to explore the effects of miR-215 expression regulation on Rb1 expression through the transfection with miR-215 mimics and inhibitors.

## Materials and Methods

### Sample collection

A total of 128 patients with Rb, treated in the Affiliated Hospital of Shaoxing University (Shaoxing, China) from July 2012 to June 2016, were selected. All patients were diagnosed with Rb. Their ages ranged from 5 months to 9 years with an average age of 4.62±2.55 years. All patients received radiotherapy and local chemotherapy before surgery. After operation, the resected specimens were divided into two parts. One part was frozen in liquid nitrogen within 30 sec, and then it was transferred to a refrigerator at −80°C, in order to be used 12 h later. The second part was sent to the Pathology Department for histological examination to confirm the diagnosis of Rb. Healthy tissues were collected within 10 mm around the tumor.

The study was approved by the Ethics Committee of the Affiliated Hospital of Shaoxing University, and the parents of all the child patients signed an informed consent.

### Main reagents and materials

RNA extraction reagent TRIzol, Lipofectamine 2000™ cell transfection liposomes and RT-PCR kit were purchased from Invitrogen (Invitrogen; Thermo Fisher Scientific, Inc., Waltham, MA, USA; 15596018, 11668030 and 10928042, respectively). Reverse transcription kit and TaqMan miRNA kit were purchased from Applied Biosystems (Applied Biosystems; Thermo Fisher Scientific, Inc.; A25576). Human Rb cells Y79 and human retinal epithelial cells APRE-19 were purchased from BeNa Culture Collection (Beijing, China; BNCC341293 and BNCC102024, respectively). Human Rb cells HXO-Rb44 were purchased from Shanghai Zishi Biotechnology Co., Ltd. (Shanghai, China; Qs101274). miR-215 expression plasmid (miR-215 mimics), miR-215 inhibitory plasmid (miR-215 inhibitor) and its corresponding control plasmid were purchased from Shanghai GenePharma Co., Ltd. (Shanghai, China). Mouse anti-human Rb1 monoclonal antibody, goat anti-mouse IgG HRP-conjugated polyclonal antibody and β -actin polyclonal antibody (cat. nos. MAB6495, HAF007, and MAB8929, respectively) were purchased from R&D Systems, Inc.

### Cell culture

Y79, HXO-Rb44 and APRE-19 cells were cultured with DMEM containing 10% fetal calf serum and 1% penicillin-streptomycin in an incubator (37°C, 5% CO 2). After subculture, cells were collected during logarithmic growth phase for subsequent experiments.

### Plasmid construction and transfection

MiR-215 mimics was transfected into Rb cells using Lipofectamine 2000 reagent and the cells were cultured in an incubator (37°C, 5% CO2) for 48 h. The medium was changed every 12 h. Then, hygromycin selection was performed. The samples were divided into miR-215 mimics group (overexpression group) and HXO-Rb44 cell group (blank group).

### Western blotting

Total protein was extracted from the cells using RIPA kit (89901), and protein concentration was measured using BCA kit (23229) (both from Thermo Fisher Scientific, Inc.). A total of 8 μl protein was added per lane and separated by 12% SDS-PAGE, and transferred to a PVDF membrane after ionization. After dyeing with Ponceau S staining solution, PBST was soaked for 5 min, and sealed for 2 h with 5% skimmed milk powder. The primary antibody was added (1:1,000) and sealed overnight at 4 °C. The primary antibody was then removed by washing, and HRP-labeled goat anti-mouse secondary antibody (1:5,000) was added, incubated at 37 °C for 1 h, and rinsed 3 times with TBST for 5 min each time. Color development was performed using ECL chemiluminescence in the dark, and the excess liquid on the membrane was dried up using filter paper. The protein bands were scanned and the gray values were analyzed using the Quantity One software (Bio-Rad Laboratories, Inc., Hercules, CA, USA): Relative expression level of protein = (gray value of the target protein band)/(gray value of the β-actin protein band). The experiment was carried out 5 times and the average values were calculated.

### Reverse transcription-quantitative PCR (RT-qPCR) detection

Total RNA was extracted from cancer and adjacent healthy tissues by TRIzol reagent. The purity of total RNA was measured by an ultraviolet spectrophotometer (α-1900splus;

Shanghai Lab-Spectrum Instruments Co., Ltd., Shanghai, China). RNA samples were stored at −80°C before using. Reverse transcription was performed to synthesize cDNA from 1 μg RNA by reverse transcription kit according to the manufacturer′s instructions. miR-215 primers were synthesized by Tiangen Biotech Co., Ltd. (Beijing, China). Primers used were: miR-215 forward, 5′-CTCGAGATGTCATCCTCAG-3′ and reverse, 5′-GAATTCGTGAGTTCTTCTG-3′; U6 forward, 5′-AGCCACATCGCTCAGACA-3′ and reverse, 5′-TGGACT CCACGACGTACT-3′. RNA template (5.0 μl), U6 reverse transcription primer (2.0 μm/l, 1.0 μl), miR-215 reverse transcription primer (2.0 μm/l, 1.0 μl), dNTPs (100 mM, 0.15 μl) were used. Denaturation was performed at 70 °C for 10 min in the PCR machine, circulation followed at 4 °C, and reverse transcription buffer (1 μl of 10X), 0.25 μl of RNase inhibitor, 0.25 μl of M-MuLV reverse transcriptase, and 3.5 μl of RNase-free water were added, at 42 °C for 60 min. cDNA was synthesized at 95 °C for 5 min. Then, the reaction was stopped. PCR reactions were performed on ABI 7900 real-time fluorescence quantitative PCR instrument (Applied Biosystems; Thermo Fisher Scientific, Inc.) according to the manufacturer’s instructions. Reaction system: cDNA 1 μl, upstream and downstream primers 0.4 μl, 2X TransStart® Top Green qPCR SuperMix (Beijing Transgen Biotech Co., Ltd., Beijing, China) 10 μl, 50X ROX Reference Dye II (Thermo Fisher Scientific, Inc.) 0.4 μl, and finally ddH2O was added to a final volume of 20 μl. Amplification conditions: 95 °C for 10 min, 95 °C for 30 sec and 60 °C for 3 min. U6 was used as internal reference. The expression values were calculated using 2−ΔΔCq method (12). The experiment was performed 5 times, and the average values were calculated.

### Statistical analysis

Statistical analyses were performed using SPSS 20.0 software package (IBM Corp., Armonk, NY, USA) and GraphPad Prism 7 software (GraphPad Software, Inc., San Diego, CA, USA). Counting data are represented by rate (%). Comparisons of count data between groups were made by χ2 test. Measurement data were expressed as mean ± standard deviation, and comparisons between groups were made using t-test. ANOVA was used for comparison among multiple groups, and the pairwise comparison after ANOVA was performed by LSD-t test. Pearson’s correlation was used to analyze the correlation between miR-215 and Rb1. *P<*0.05 was considered to indicate a statistically significant difference.

## Results

### Clinical data of patients

A total of 128 children with Rb were enrolled in this study. The average age of the children was 4.62±2.55, including 49 males and 79 females, 82 cases of nerve infiltration and 46 cases of non-infiltration, 51 cases of lymphatic metastasis and 77 cases of non-metastasis, 38 cases of differentiation and 90 cases of undifferentiation, 55 cases of monocular involvement and 73 cases of binocular involvement.

### Expression of miR-215 in tissues and cells

**Fig. 1:**
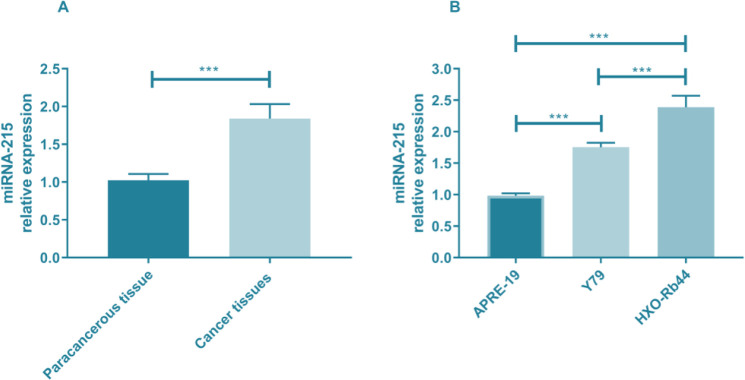
Expression of miR-215 in tissues and cells. A. miR-215 expression in tissues. B. Expression of miR-215 in cells. ***P<0.001

We detected the miR-215 expression in cancer and adjacent tissues of patients, and found that the expression of miR-215 in the adjacent tissues was significantly lower than that in the cancer tissues (t=45.693, *P<*0.001). In addition, the expression of miR-215 was detected in Y79, HXO-Rb44 and APRE-19 cells, and it was found that there were significant differences among the three groups (F=111.267, *P<*0.001). The expression of miR-215 in Y79 and HXO-Rb44 cells was significantly higher than that in APRE-19 cells (*P<*0.001), and in HXO-Rb44 cells was significantly higher than that in Y79 cells (*P<*0.001) (Fig. 1).

### Association of miR-215 expression with the clinical factors of Rb

Patients were divided into high miR-215 expression group (≥median value) and low miR-215 expression group (<median value), according to the median value of miR-215 expression. The analysis of the results showed that miR-215 expression has no significant association with sex, age, lymph node metastasis and number of affected eyes (*P>*0.05), but is significantly associated with the nerve infiltration (*P<*0.001) and the degree of differentiation (*P<*0.020) (Table 1).

**Table 1:** Association of miR-215 expression with the clinical factors of Rb patients (n=128)

***Factor***		***n***	***miR-215 expression***		***X^2^***	***P***
			***High expression (n=64)***	***Low expression (n=64)***		
Sex					0.525	0.46
	Male	49	23 (35.94)	27 (42.19)		
	Female	79	41 (64.06)	37 (57.81)		
Age(yr)					3.153	0.076
	<4	70	40 (62.5)	30 (46.88)		
	≥4	58	24 (37.5)	34 (53.13)		
Nerve invasion					10.995	<0.001
	Yes	82	32 (50)	50 (78.13)		
	No	46	32 (50)	14 (21.88)		
Lymph node metastasis					0.815	0.367
	Yes	51	28 (43.75)	23 (35.94)		
	No	77	36 (56.25)	41 (64.06)		
Differentiation degree						
	differentiation	38	13 (20.31)	25 (39.06)	5.390	0.020
	Undifferentiated	90	51 (79.69)	39 (60.94)		
Number of affected eyes						
	Monocular	55	26 (40.63)	29 (45.31)	0.051	0.821
	Both eyes	73	38 (59.38)	35 (54.69)		

Rb, retinoblastoma.

### Expression of Rb1 in tissues and cells

**Fig. 2:**
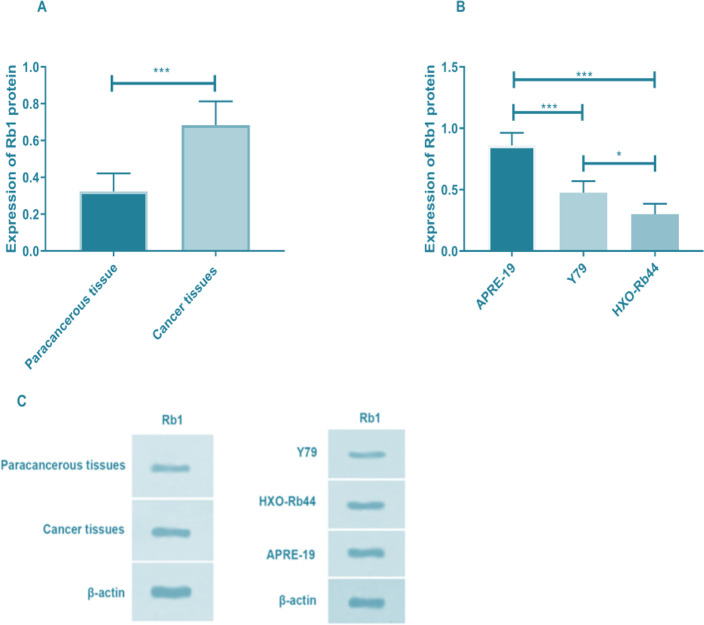
Expression of Rb1 protein in tissues and cells. A. Expression of Rb1 protein in tissues. B. Expression of Rb1 protein in tissues in a cell line. C.WB diagram. *P<0.05, ***P<0.001

We first detected the expression of Rb1 protein in cancer tissues and found that the expression of Rb1 was significantly lower than that in adjacent tissues (*P<*0.001). The expression of Rb1 protein in the three groups of cells was also significantly different (F=42.352, *P<*0.05). In APRE-19 cells, the Rb1 protein expression was the lowest, and statistically different from Y79 and HXO-Rb44 cells (*P<*0.001). In HXO-Rb44 cells, the expression of Rb1 was significantly higher than that in Y79 cells (*P<*0.05) (Fig. 2 and Table 2).

**Table 2:** Expression of Rb1 in tissues and cells

***Type***	***Expression of*** Rb1 protein	***t/F value***	***P value***
Tissues			
Cancer tissues	0.324±0.097	25.235	<0.001
Adjacent tissues	0.684±0.129^a^		
Cells			
APRE 19	0.348±0.072	42.352	<0.001
Y79	0.702±0.094^b^		
HXO Rb44	0.861±0.102^b,c^		

a *p<*0.05, compared with cancer tissues;

b *p<*0.05, compared with APRE-19 cells;

c *p<*0.05, compared with Y79 cells. Rb, retinoblastoma

### Expression of miR-215 and Rb1 proteins in cells after transfection

**Fig. 3:**
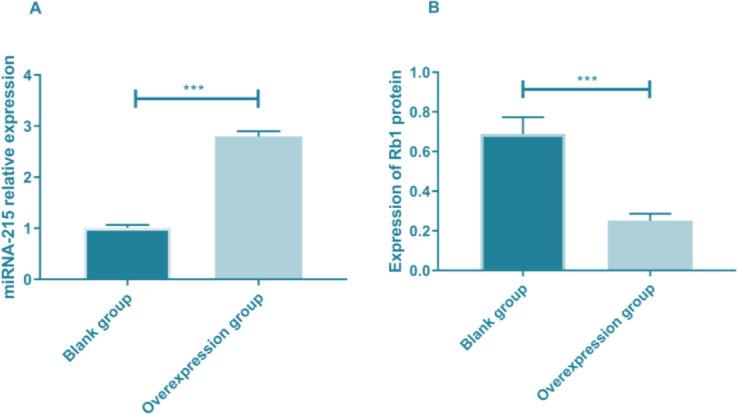
Expression of miR-215 and Rb1 protein after transfection. A. QRT-PCR was used to detect the expression of miR-215 in cells after transfection. B.WB detection of Rb1 protein expression in cells after transfection

HXO-Rb44 cells were divided into overexpression and blank group according to the expression of miR-215 and Rb1 protein. The expression of miR-215 in the overexpression group was significantly higher than that in blank group (*P<*0.001). The expression of Rb1 protein in blank group was significantly higher than that in the overexpression group (*P<*0.001) (Fig. 3 and Table 3).

**Table 3:** Expression of miR-215 and Rb1 proteins in cells after transfection

***Group***	***Relative expression*** of miR 215	***Expression of*** Rb1 protein
Blank group	1.010±0.055	0.689±0.084
Overexpression group	2.795±0.102	0.251±0.035
*t* value	34.443	10.763
*P* value	<0.05	<0.05

Rb, retinoblastoma.

### Correlation analysis of miR-215 and Rb1 protein in tissues

**Fig. 4:**
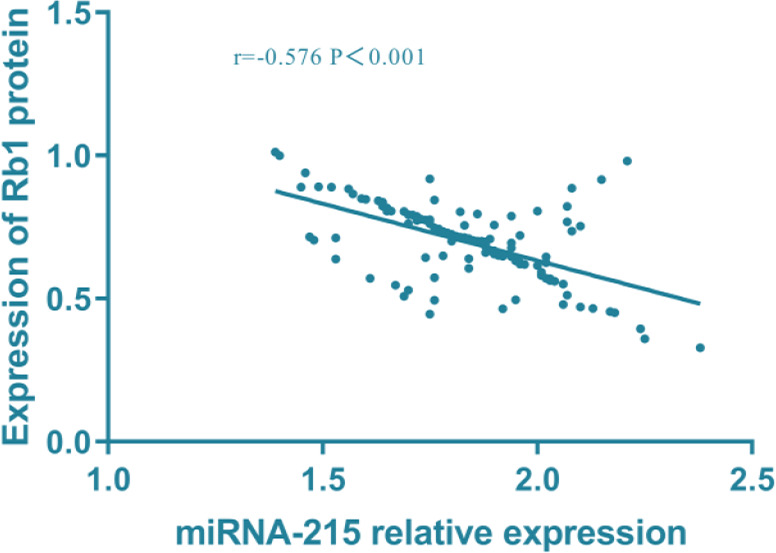
Correlation analysis of miR-215 and Rb1 in tissues

Pearson test was used to analyze the correlation between miR-215 and Rb1 in tissues. There was a negative correlation between miR-215 and Rb1 in tissues of patients, and Rb1 expression decreased with the increase of miR-215 (r=−0.576, *P<*0.001) (Fig. 4).

## Discussion

Rb originates from retinal embryonic nucleus cells and the survival rate of patients with this disease is 63% in China (13), which is lower than that in developed countries (95%) (14). Rb is a common type of malignant tumor in the eyes with unclear pathogenesis. Approximately 6% of this disease is caused by autosomal dominant gene inheritance, and most of the cases are sporadic. About 1/4 of this disease is caused by gene mutation, and in the rest of the cases is caused by cell mutations. However, some scholars believe that this disease is also correlated with viral infection and other reasons. The occurrence of this disease is significantly correlated with the loss and inactivation of Rb alleles (8,15,16).

In recent years, miRNAs were important biomarkers for the diagnosis of different diseases. miRNAs are usually differentially expressed in tumor tissues, and can be divided into two major categories according to their function, namely, oncogenes and tumor suppressor genes (17). miR-215 is located on human chromosome 1q41 and is expressed in rectal, gastric and kidney cancer. miR-215 regulates the invasion and proliferation of cancer cells by regulating the expression of target genes (18). As an important tumor suppressor gene, the low expression level of Rb1 is an important signal of Rb. The activation of Rb1 signal can inhibit the development of tumors.

In this study, we first examined the relative expression of miR-215 in Rb tissues and found that the expression of miR-215 was significantly higher in cancer tissues than in adjacent tissues, which indicated that the expression of miR-215 in tissues is significantly increased after Rb occurs in children. Up to our knowledge, no studies have previously shown that miR-215 is expressed in Rb tissues. This study is the first to confirm the high expression of miR-215 in Rb tissues, and the same high expression of miR-215 was also found in Y79, and HXO-Rb44 cells, which is a good confirmation of our research results. Rb signaling pathway plays an important role in the process of tumor suppression and can effectively inhibit cell proliferation. Overexpression of miR-215 can target the downregulation of the expression of Rb1 protein in gliomas and affect cell proliferation and invasion (19). However, it is not clear whether there is the same effect in Rb. Therefore, we detected the relative expression of Rb1 protein in cancer tissues and cells and found that the expression of Rb1 protein in cancer tissues was significantly lower than that in adjacent tissues, and the relative expression of Rb1 protein in Y79 and HXO-Rb44 cells was also lower than that in APRE-19 cells, suggesting that miR-215 may also regulate the expression of Rb1 protein in Rb. Furthermore, we overexpressed the miR-215 mimics of HXO-Rb44 cells and found that the relative expression of Rb1 protein in the overexpression group was significantly decreased, indicating that miR-215 can also inhibit Rb1 protein in Rb cells. Finally, we analyzed the correlation between the relative expression of miR-215 and the relative expression of Rb1 protein in the overexpressing cell lines, and found a negative correlation between them.

In this study, the expression of Rb1 protein in Rb cells was significantly downregulated by the up-regulation of miR-215, and the expression level of Rb1 protein was increased by the downregulation of miR-215. This suggests that miR-215 can affect the development and progression of Rb by inhibiting the expression of Rb1 protein in cells. Over the past decade, with the continuous improvement of treatment methods, the requirement of Rb treatment is not only to save the lives of patients, but also to save the eyes and vision, and to improve the quality of life of the patients and their families. At present, the most effective conservative treatment is the chemical reduction method. It is economically and practically effective, and can be used to reduce the burden caused to the patients and their families. However, early diagnosis of Rb can effectively reduce the unnecessary loss.

This study is still challenged by some shortcomings. Firstly, we have not further tested the biological function of cells after overexpression. Secondly, the small number of samples may have some impact on the results of the study. Therefore, we hope to increase the number of samples in future research to support the results of this study.

## Conclusion

The expression level of miR-215 was significantly increased in Rb tissues compared with normal tissues, and was correlated with the degree of differentiation and nerve infiltration. Increased expression of miR-215 can inhibit the expression of intracellular Rb1 protein. The results of this study suggest that miR-215 can potentially serve as a biomarker for the diagnosis and prognosis of Rb, or even a target for the treatment of this disease.

## Ethical considerations

Ethical issues (Including plagiarism, informed consent, misconduct, data fabrication and/or falsification, double publication and/or submission, redundancy, etc.) have been completely observed by the authors.
